# Risk factor profile and outcomes of premature acute coronary syndrome after percutaneous coronary intervention: A 1‐year prospective design

**DOI:** 10.1002/clc.24170

**Published:** 2023-10-11

**Authors:** Aida Fallahzadeh, Saghar Mehraban, Tara Mahmoodi, Ali Sheikhy, Mohammadreza Naderian, Phd Afsaneh Aein, Hamed Rafiee, Mehdi Mehrani, Masih Tajdini, Farzad Masoud‐kabir, Saeed Sadeghian, Kaveh Hosseini

**Affiliations:** ^1^ Tehran Heart Center, Cardiovascular Disease Research Institute Tehran University of Medical Sciences Tehran Iran; ^2^ Mayo Clinic Rochester Minnesota USA; ^3^ School of Medicine Isfahan University of Medical Sciences Isfahan Iran; ^4^ Cardiac Primary Prevention Center, Cardiovascular Diseases Research Institute, Tehran University of Medical Sciences Tehran University of Medical Sciences Tehran Iran

**Keywords:** acute coronary syndrome, outcome, percutaneous coronary intervention, premature coronary artery disease

## Abstract

**Background:**

The prevalence of acute coronary syndrome (ACS) among young adults (premature ACS) has dramatically increased in recent years, especially in developing countries. Yet, the data on these patients' attributed risk factors and outcomes are inconsistent. In this study, we aimed to investigate these data in a cohort of premature ACS cases who underwent percutaneous coronary intervention (PCI) compared to older patients.

**Hypothesis:**

We hypothesize that premature ACS patients undergoing PCI will exhibit different risk factor profiles and outcomes compared to non‐premature patients. specifically, we anticipate that premature patients do not necessarily have better outcomes than non‐premature.

**Methods:**

Overall, 3142 and 10 399 patients were included in premature and non‐premature groups, respectively. Patients' pre‐operative, post‐operative, and follow‐up data were retrieved retrospectively from the Tehran Heart Center PCI databank.

**Results:**

The mean age of premature and non‐premature cohorts was 48.39 and 67 years, respectively. Patients were predominantly male in both groups. Family history of coronary artery disease (CAD), dyslipidemia, smoking, and opium addiction were more prevalent among the younger cohort. After adjustment, in‐hospital mortality in younger patients was considerably higher, with all‐cause mortality and major cardiovascular and cerebrovascular events (MACCE) exhibiting no noticeable difference among the two groups.

**Conclusions:**

Risk factor profile is different in young patients, and traditional cardiovascular risk factors, such as hypertension and diabetes mellitus, are more prevalent among older adults. Younger age is not equivalent to a better prognosis; hence, similar or even more caution should be taken into consideration regarding secondary prevention for these patients.

## Graphical abstract

Our study compared risk factors, all‐cause mortality, and major cardiovascular and cerebrovascular events in premature and nonpremature patients who underwent percutaneous coronary intervention. Younger patients exhibited distinct risk factors, with higher in‐hospital mortality but similar long‐term outcomes. This highlights the need for caution in secondary prevention for younger patients.

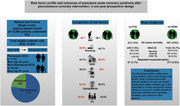

## 1 INTRODUCTION

Premature acute coronary syndrome (ACS) is defined as the first occurrence of clinical manifestation of atherosclerosis in men and women at a young age.^1^ Studies show that the mean age of coronary artery disease occurrence has decreased in recent years especially in Iran, mainly due to an increase in urbanization and unhealthy lifestyles.^2^, ^3^ According to the Global Burden of Diseases study, ischemic heart disease is one of the important causes of morbidity and mortality among the young population, especially in developing countries.^4^, ^5^, ^6^

The prevalence of premature ACS varies from one country to another; all healthcare providers should, therefore, evaluate the associated risk factors and mortality and morbidity rates to improve the general health of the population. Risk factors, diabetes mellitus, family history of coronary artery disease, dyslipidemia, smoking, and hypertension were significantly and positively associated with coronary artery disease in young adults compared to healthy age‐ and sex‐matched populations in Iran.^7^

Little evidence exists regarding the association between age and adverse outcomes among patients with ACS undergoing percutaneous coronary intervention (PCI),^8^ such information can help healthcare systems to achieve better planning and apply preventive strategies for improving clinical outcomes following PCI to plan and manage coronary artery disease more properly. Thus, we aimed to compare the outcomes among the patients with ACS who underwent PCI, 1 year after the procedure in both men and women; collected data from the PCI data bank at the Tehran Heart Center (THC) over a span of 4 years with reliable follow‐ups.

## 2 MATERIALS AND METHODS

### 2.1 Study design

This study was a single‐center, registry‐based cohort study conducted at the THC.^9^ Data regarding the patient's characteristics and procedural details were recorded prospectively at the time of the procedure and during each follow‐up afterward. The THC ethical board approved the study (IR‐THC‐13 799). This study did not meet the criteria for informed consent; consequently, an “informed consent waiver” was obtained from the THC ethical board.

### 2.2 Study population

The entire cohort included 15 809 patients who underwent coronary angiogram and PCI at the THC between April 2015 and December 2019. Our inclusion criteria were: (1) all patients with ACS (see after for definition); (2) adequate preprocedural characteristics; and (3) completion of 1‐year follow‐up visits. The main exclusion criteria were patients presenting with stable angina and incomplete follow‐ups. The patients' follow‐up protocol was at the 4th, 6th, and 12th month after PCI and annually thereafter. Patients were stratified into two groups; Premature ACS (women ≤55 years; men ≤50 years) and nonpremature ACS (women >55 years; men >50 years), based on the time of the procedure. Patients did not switch groups if they passed the cut‐off points during the follow‐up period.

### 2.3 Definition of variables

Data from the study population were retrieved from our center's angiography/PCI databank.

ACS refers to a spectrum of clinical presentations ranging from those for ST‐elevated myocardial infarction (STEMI) to presentations found in non‐ST‐elevated myocardial infarction (NSTEMI) or in unstable angina (UA). The definition of hypertension was based on the presence of at least one of the following conditions; minimum systolic blood pressure of 140 mmHg, minimum diastolic blood pressure of 90 mmHg, or a history of taking antihypertensive drugs.^10^ The definition of dyslipidemia was based on the presence of either a minimum total cholesterol of 240 mg/dL, a minimum triglyceride of 200 mg/dL, or high‐density lipoprotein cholesterol <40 mg/dL in men and <50 mg/dL in women, a minimum low‐density lipoprotein cholesterol level of 160 mg/dL, or a history of taking lipid‐lowering drugs.^11^, ^12^ We determined cigarette smoking and opium consumption based on the patient's self‐reported status. A current smoker was a person who currently smokes and has ever smoked more than 100 cigarettes.^13^ Based on the American Diabetes Association, diabetes mellitus was defined as the presence of a definite history of diabetes with records of treatment or fasting blood sugar ≥126 mg/dL or 2‐h postprandial glucose ≥200 mg/dL.^14^ Positive family history was defined as a first‐degree relative younger than 55 years (men) or 65 years (women) who had coronary artery disease.

We used thrombolysis in myocardial infarction (TIMI) flow grade to assess the coronary artery flow in ACS. It is classified as grade 0 (no flow), grade 1 (penetration without perfusion), grade 2 (partial perfusion), or grade 3 (complete perfusion). Left main (LM) stenosis refers to those with stenosis of the left main coronary artery >50% on angiogram.

#### 2.3.1 Procedural technique

The standard techniques with the femoral approach were applied for performing PCI procedures. According to our procedure routine, all patients received a 300–600 mg loading dose of clopidogrel plus 325 mg aspirin before the procedure. During the procedure, patients received 70–100 IU/kg intravenous unfractionated heparin. Clopidogrel (75 mg/day) and aspirin (81 mg/day) were maintained for at least 1‐year after the procedure.

#### 2.3.2 Study endpoints

All‐cause mortality is defined as the occurrence of death of any cause during the follow‐up, in‐hospital mortality is defined as the occurrence of death during the first 30 days after the procedure, and MACCE occurrence is defined as the occurrence of one of the following conditions (which has happened first): all‐cause mortality, nonfatal ACS, nonfatal stroke or transient ischemic attack, and repeat coronary revascularization via PCI or Coronary artery bypass graft surgery were considered as main endpoints.

### 2.4 Statistical analysis

Normally and skewed distributed continuous variables were presented as mean with standard deviation (SD) or median with 25th and 75th percentiles (interquartile range [IQR] boundaries), respectively. Student's *t*‐test and Mann–Whitney *U*‐test were used to compare normally and skewed distributed variables, respectively. Discrete variables were presented as frequency and percentages and compared between groups using the *χ*² test. The unadjusted in‐hospital mortality was reported using logistic regression (odds ratio [OR]). The unadjusted all‐cause mortality and MACCE were reported using Cox's proportional hazards model (hazard ratios [HR]).

Inverse probability weights (IPW) were used to stabilize potential selection biases of treatment, weights were calculated from propensity score (PS), which was generated by predicted probabilities of logistic regression on identified potential confounders (gender, body mass index [BMI], hemoglobin [Hb], high‐density lipoprotein [HDL], estimated glomerular filtration rate [eGFR], triglyceride [TG], low‐density lipoprotein [LDL], ejection fraction [EF], presenting first diagnosis, total cholesterol [TCH], family history, dyslipidemia [DLP], diabetes mellitus [DM], hypertension [HTN], cigarette smoking [CS], opium, cerebrovascular disease, chronic obstructive pulmonary disease [COPD], pre‐ and postprocedural TIMI flow, vessel severity [SVD, 2VD, 3VD]). The C‐statistic for the model was 0.81. Weights for each case (Wi) are calculated as 1/PS(Xi) for premature ACS, and 1/(1‐PS(Xi)) for nonpremature ACS.

We used the R version 4.0.3 for all statistical analyses.^15^

## 3 RESULTS

### 3.1 Study population

**Figure 1:**
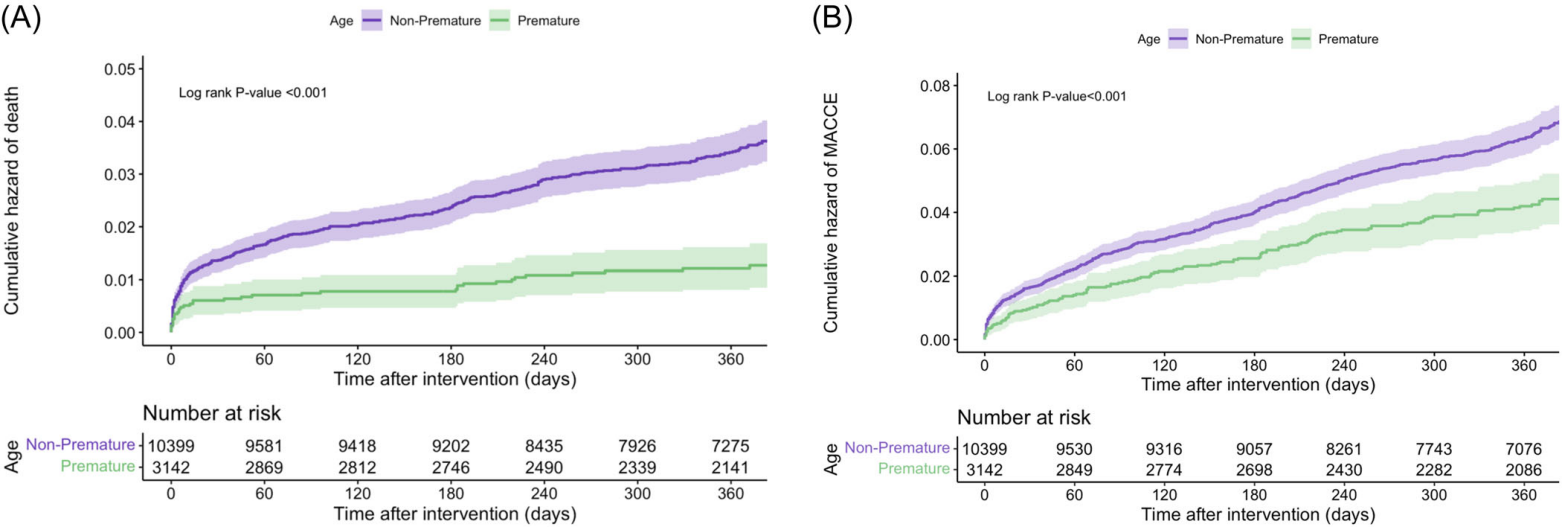
Cumulative hazard of (A) mortality and (B) major cardiovascular and cerebrovascular events (MACCE) in premature and nonpremature patients.

In all, 15 809 patients were recruited in this study. After applying exclusion criteria, 13 541 patients remained in the final analysis (Figure 1). From these, 3142 were categorized as premature ACS, and the rest (10 399) as nonpremature ACS.

The mean age of premature and nonpremature ACS groups was 48.39 ± 9.13 and 67.00 ± 12.60 years, respectively. The patients were mainly male in both groups (92.6% in premature and 66.6% in nonpremature; *p* < .001). The main presentations of ACS were unstable angina and NSTEMI in both groups (57.2% in the premature group and 65.8% in the nonpremature group; *p* < .001). In the premature group, smoking and opium consumption were higher and also, and HTN and DM were lower compared to the nonpremature group. Baseline LDL was significantly higher in younger patients.

As expected, a positive family history of CAD was higher in the premature ACS group. Detailed baseline characteristics are described in Table 1.

**Table 1:** Baseline characteristics in premature and nonpremature acute coronary syndrome (ACS) patients.

	Total	Premature (*N* = 3142)	Nonpremature (*N* = 10 399)	*p*‐Value
Age (year)	62.67 (10.99)	48.39 (5.33)	67 (8.27)	<.001
BMI (kg/m^2^)	28.27 (4.51)	28.66 (4.38)	28.15 (4.54)	<.001
Gender				<.001
Female	3708 (27.4%)	232 (7.4%)	3476 (33.4%)	
Male	9833 (72.6%)	2910 (92.6%)	6923 (66.6%)	
Hb (g/dl)	14.69 (1.82)	15.46 (1.67)	14.45 (1.80)	<.001
EF (%)	45.67 (9.13)	46.35 (8.52)	45.46 (9.30)	<.001
HDL (mg/dL)	38.93 (9.73)	36.96 (9.00)	36.52 (9.86)	<.001
eGFR (mL/min/1.73m^2^)	85.9 [66.2‐108.6]	115.06 [113.63‐116.19]	78.18 [77.65‐78.82]	<.001
LDL (mg/dL)	98.29 (35)	149 [146‐152]	127 [126‐129]	<.001
TG (mg/dL)	155.34 (101.23)	102 [101‐104]	92 [92, 93]	<.001
NSTEMI/UA	8640 (63.8%)	1796 (57.2%)	6844 (65.8%)	<.001
DM	5421 (40%)	953 (30.3%)	4468 (43%)	<.001
HTN	7258 (53.6%)	1089 (34.7%)	6169 (59.3%)	<.001
DLP	8237 (60.8%)	1962 (62.4%)	6275 (60.3%)	.034
CS				<.001
Current	4014 (29.6%)	1480 (47.1%)	2534 (24.4%)	
Former	1556 (11.5%)	273 (8.7%)	1283 (12.3%)	
Passive	139 (1%)	19 (0.6%)	120 (1.2%)	
Opium				<.001
Current	1474 (10.9%)	405 (12.9%)	1069 (10.3%)	
Former	618 (4.6%)	243 (7.7%)	375 (3.6%)	
Family history	2639 (19.5%)	927 (29.5%)	1712 (16.5%)	<.001
Cerebrovascular disease	432 (3.2%)	35 (1.1%)	397 (3.8%)	<.001
COPD	308 (2.3%)	36 (1.1%)	272 (2.6%)	<.001
Congestive heart failure	369 (2.7%)	49 (1.6%)	320 (3.1%)	<.001
Peripheral vascular disease	41 (0.3%)	6 (0.2%)	35 (0.3%)	.193
Renal failure	222 (1.6%)	19 (0.6%)	203 (2%)	<.001
Coronary artery disease history	6706 (49.5%)	1302 (41.4%)	5404 (52%)	<.001
Vessel severity				<.001
SVD	4941 (36.4%)	1415 (45%)	3526 (33.8%)	
2VD	4658 (34.4%)	1063 (33.8%)	3595 (34.6%)	
3VD	3942 (29.1%)	664 (21.1%)	3278 (31.5%)	
Left main artery stenosis	369 (2.7%)	40 (1.3%)	329 (3.2%)	<.001
Door‐to‐needle time (min)	54 [37–89]	53 [50–55]	55 [55–58]	.247
Preprocedural TIMI flow	1.98 (1.29)	1.77 (1.33)	2.05 (1.26)	<.001
Postprocedural TIMI flow	2.93 (0.38)	2.94 (0.36)	2.93 (0.39)	.214

Abbreviations: 2VD, two vessel disease; 3VD, three vessel disease; BMI, body mass index; COPD, chronic obstructive pulmonary disease; CS, cigarette smoking; DLP, dyslipidemia; DM, diabetes mellitus; EF, ejection fraction; eGFR, estimated glomerular filtration rate; FH, family history; Hb, hemoglobin; HDL, high density lipoprotein; HTN, hypertension; LDL, low density lipoprotein; NSTEMI, non‐ST elevation myocardial infarction; SVD, single vessel disease; TG, triglyceride; TIMI, thrombolysis in myocardial infarction; UA, unstable angina.

### 3.2 Clinical outcomes

#### 3.2.1 Total population

The absolute number of all‐cause mortalities was 52 (1.6%) and 507 (4.8%) in the premature and nonpremature ACS patients, respectively (Table 2). MACCE occurred in 238 (7.5%) patients with premature ACS and 1125 (10.7%) with nonpremature ACS, The Breakdown of the MACCE outcomes was as follows; Mortality occurred in 48 (1.5%) of premature patients and in 453 (4.3%) of nonpremature patients. MI/ACS events happened in 101 (3.2%) of premature patients and 367 (3.5%) of nonpremature patients. Additionally, CVA/TIA occurred in 2 (0.1%) of premature patients and 28 (0.3%) of nonpremature patients.

**Table 2:** Rates of post‐percutaneous coronary intervention (PCI) outcome in premature and nonpremature ACS groups.

	Premature (*n* = 3142)	Nonpremature (*n* = 10 399)
All‐cause mortality	52 (1.6%)	507 (4.8%)
In‐hospital mortality	14 (0.4%)	118 (1.1%)
MACCE	238 (7.5%)	1125 (10.7%)
Mortality	48 (1.5%)	453 (4.3%)
MI/ACS	101 (3.2%)	367 (3.5%)

Abbreviations: ACS, acute coronary syndrome; MACCE, major adverse cerebro‐cardiovascular events; MI, myocardial infarction.

Composite 1‐year outcomes (all‐cause mortality, MAACE, and in‐hospital mortality) were significantly lower in the premature group using an unadjusted cox‐regression model (HR = 0.342, 95% confidence interval [CI] [0.252–0.465], *p* < .001; HR = 0.728, 95% CI [0.631–0.840], *p* < .001; and HR = 0.343, 95% CI [0.143–0.697], *p* = .007 respectively). However; after matching (with an adjusted IPW‐based analysis), no significant difference was observed between all‐cause mortality and MACCE of the premature and nonpremature ACS groups. However, In‐hospital mortality was significantly higher in the premature group (HR = 1.041, 95% CI [0.371–2.922], *p* = .939; HR = 0.984, 95% CI [0.588–1.646], *p* = .952; and HR = 2.848, 95% CI [2.264–3.583], *p* < .001, respectively) (Table 3) (Figure 1).

**Table 3:** Comparison between premature and nonpremature post‐percutaneous coronary intervention (PCI) outcomes.

Outcome	All‐cause mortality		Major cardiovascular and cerebrovascular events (MACCE)		In‐hospital mortality	
Adjustment	Unadjusted	Inverse probability weights (IPW)	Unadjusted	IPW	Unadjusted	IPW
Nonpremature	Ref.	Ref.	Ref.	Ref.	Ref.	Ref.
Premature	0.342 (0.252–0.465), *p* < .001	1.041 (0.371–2.922), *p* = .939	0.728 (0.631–0.840) *p* < .001	0.984 (0.588–1.646) *p* = .952	0.343 (0.143–0.697) *p* = .007	2.848 (2.264–3.583) *p* < .001

#### 3.2.2 Based on gender (male vs. female)

**Figure 2:**
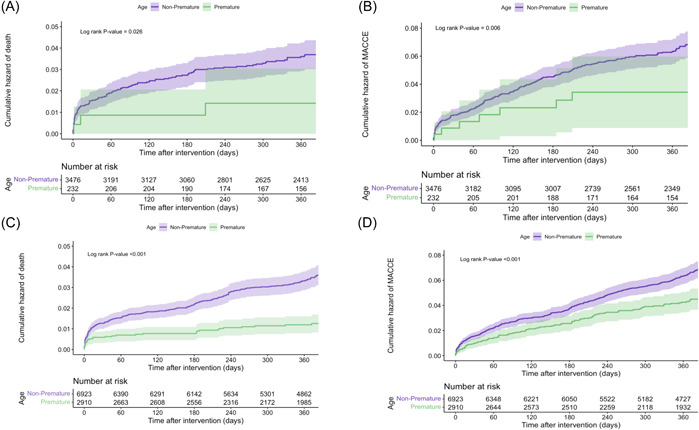
Cumulative hazard of (A) mortality in women, (B) major cardiovascular and cerebrovascular events (MACCE) in women, (C) mortality in men, and (C) MACCE in men.

In men, all‐cause mortality and MACCE were statistically similar in the premature and nonpremature group however in‐hospital mortality was higher in younger patients, HR 4.232 (3.284–5.521) (*p* < .001). In women, although all‐cause mortality was lower in premature patients, the MACCE was not different HR 0.762 (0.290–1.999) (*p* = .581) (Table 4) (Figure 2).

**Table 4:** Comparison between premature and nonpremature post‐percutaneous coronary intervention (PCI) outcomes according to sex.

Outcome		All‐cause mortality		Major cardiovascular and cerebrovascular events (MACCE)		In‐hospital mortality	
Adjustment		Unadjusted	Inverse probability weights (IPW)	Unadjusted	IPW	Unadjusted	IPW
Women	Nonpremature	Ref.	Ref.	Ref.	Ref.	‐	‐
	Premature	0.212 (0.052–0.858) *p* = .029	0.057 (0.013–0.255) *p* < .001	0.4 (0.2–0.777) *p* = .006	0.762 (0.290–1.999) *p* = .581	NA	NA
Men	Nonpremature	Ref.	Ref.	Ref.	Ref.	Ref.	Ref.
	Premature	0.356 (0.259–0.491) *p* < .001	1.533 (0.532–4.415) *p* = .428	0.739 (0.635–0.860) *p* < .001	1.061 (0.588–1.919) *p* = .848	0.424 (0.173–0.890) *p* = .0368	4.232 (3.284–5.521) *p* < .001

#### 3.2.3 Based on clinical presentation

**Figure 3:**
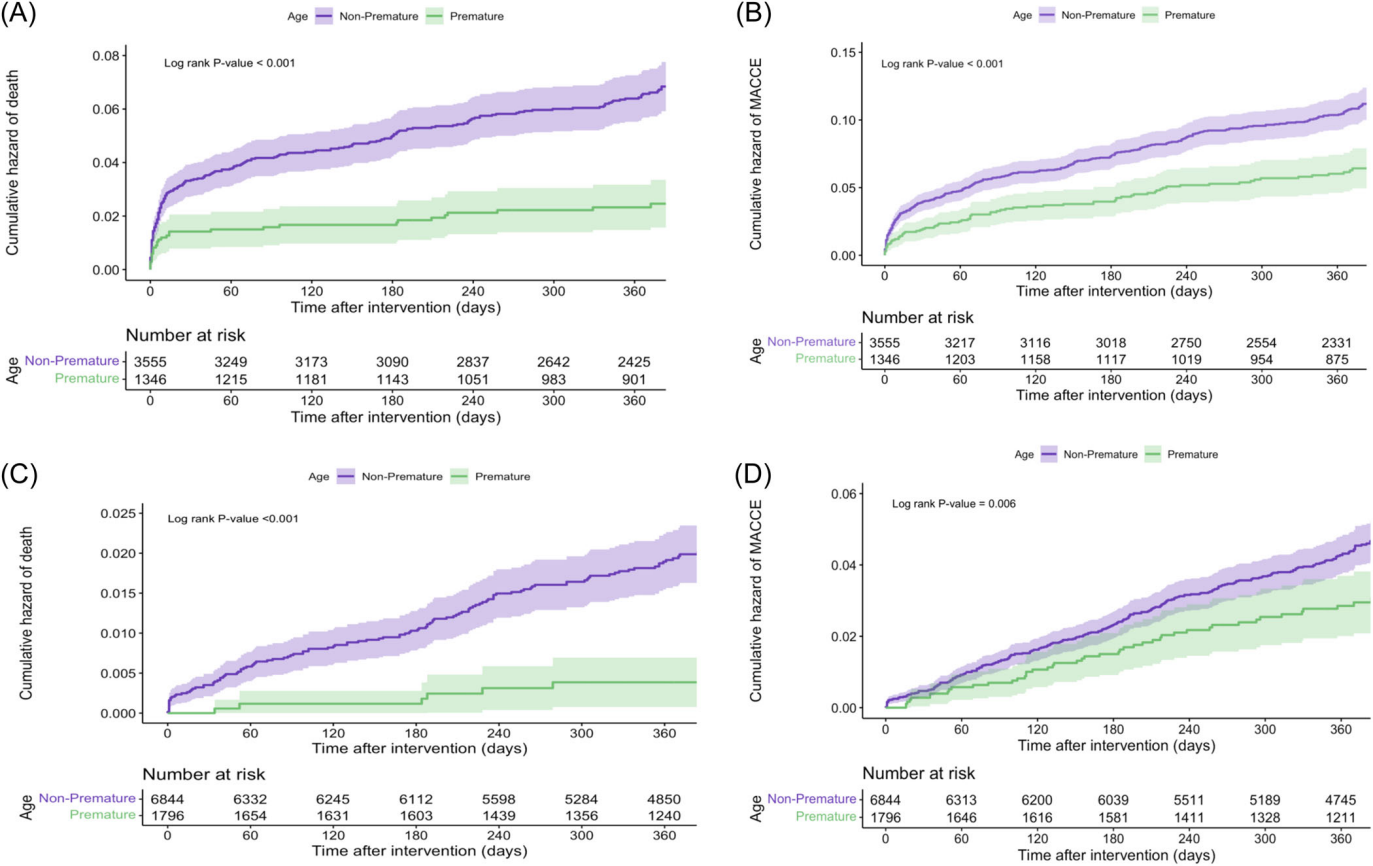
Cumulative hazard of (A) mortality in non‐ST‐elevated myocardial infarction (NSTEMI)/unstable angina (UA), (B) major cardiovascular and cerebrovascular events (MACCE) in NSTEMI/UA, (C) mortality in ST‐elevated myocardial infarction (STEMI), and (D) MACCE in STEMI.

After applying an adjusted IPW‐based model, all‐cause mortality showed no significant difference between the premature and nonpremature ACS groups neither in the patients with STEMI nor NSTEMI/UA (HR = 1.751, 95% CI [0.454–6.749], *p* = .416 and HR = 0.450, 95% CI [0.165–1.228], *p* = .119, respectively). However, MACCE was still significantly lower in the patients with premature ACS presenting with NSTEMI/UA in the adjusted model (HR = 0.532, 95% CI [0.356–0.796], *p* = .002). In‐hospital mortality was significantly higher in the premature ACS patients who presented with STEMI (HR = 4.279, 95% CI [3.373–5.478], *p* < .001) (Table 5) (Figure 3).

**Table 5:** Comparison between premature and nonpremature post‐percutaneous coronary intervention (PCI) outcomes according to acute coronary syndrome (ACS) presentation.

Outcome		All‐cause mortality		Major cardiovascular and cerebrovascular events (MACCE)		In‐hospital mortality	
Adjustment		Unadjusted	Inverse probability weights (IPW)	Unadjusted	IPW	Unadjusted	IPW
ST‐elevated myocardial infarction (STEMI)	Nonpremature	Ref.	Ref.	Ref.	Ref.	‐	‐
	Premature	0.324 (0.220–0.477) *p* < .001	1.751 (0.454–6.749) *p* = .416	0.663 (0.540–0.813) *p* < .001	1.669 (0.773–3.603) *p* = .192	0.329 (0.136–0.676) *p* = .005	4.279 (3.373–5.478) *p* < .001
Non‐ST‐elevated myocardial infarction (NSTEMI)/unstable angina	Nonpremature	Ref.	Ref.	Ref.	Ref.	Ref.	Ref.
	Premature	0.309 (0.186–0.514) *p* < .001	0.450 (0.165–1.228) *p* = .119	0.755 (0.618–0.924) *p* = .006	0.532 (0.356–0.796) *p* = .002	NA	NA

## 4 DISCUSSION

Based on our definition, roughly a quarter of ACS patients were premature. Smoking habits, positive family history of CAD, Baseline LDL levels, and opium addiction were higher in the premature group. However, other major traditional CAD risk factors, such as HTN and DM, were relatively lower. The adjusted in‐hospital mortality was surprisingly higher in the young cohort, with all‐cause mortality and MACCE exhibiting no difference among the two patient groups. These findings suggest that younger age is not necessarily associated with better post‐revascularization outcomes.

### 4.1 Risk factor distribution

The significant implication of smoking in premature ACS has been shown in previous literature.^16^ About half of the young population were smokers, which was almost twice the percentage of older patients (24.4%). Our results were in line with previous studies.^17^ Smoking is considered the strongest modifiable factor associated with premature CAD and ACS.^16^, ^18^ Moreover, Alberty et al. highlighted a 10‐year reduction in the age at which cardiovascular events occur due to smoking.^19^ Hence, addressing such risk factors in young people and taking proper measures to promote their cessation would yield the maximum harvest.

A family history of CAD is another traditional risk factor that is more prevalent among young patients. It is mediated by genetic determinants and diseases such as heterozygote familial hypercholesterolemia, whose prevalence is shown to be as high as 1 in 11 patients with premature ACS.^20^, ^21^ As well as this, a shared lifestyle has probably led parents to cardiovascular morbidities. The integral role that the family history of CAD plays warrants further gene identification and risk stratification in primary prevention for at‐risk populations.

Although implicated in all age groups, dyslipidemia and high LDL level were more prevalent among young patients than their older counterparts. Lipid abnormalities enhance atherosclerotic plaque formation, which can subsequently destabilize, initiating myocardial infarction (MI) and stroke field.^22^ The incidence of MACCE is substantially higher among premature patients with familial hypercholesterolemia.^20^ High levels of lipoprotein a (Lpa) are detected in young patients presenting with ACS, and its negative cardiovascular effects are shown to be aggravated in association with elevated LDL levels field.^23^ Previous studies have also demonstrated a strong association between LDL/HDL ratio and the rate of MI, especially among young patients,^16^ necessitating relevant screening and utilizing lipid‐lowering medications before cardiovascular events.

Consistent with previous studies, conventional risk factors, including HTN and DM, exhibited higher rates among the mature ACS cohort.^16^, ^24^ Yet, part of this lower prevalence among younger patients could be due to the under‐diagnosis of these conditions due to poor screening in younger age groups. Some studies have suggested utilizing HbA1C measurement as a complementary to the self‐reported history of DM in ACS patients^25^ and have revealed a direct association between elevated HbA1C level and MACCE.^26^

### 4.2 Outcomes: total population

Unadjusted analysis of our data exhibited lower rates of MACCE and all‐cause mortality within the premature cohort, which turned insignificant after adjustment. On the contrary, In‐hospital mortality increased in the premature group after adjustment. However, caution is needed in interpreting this finding according to sample size limitations and the potential influence of various confounders. Factors such as previous medical treatment, genetic predisposition, lifestyle, recreational habits, and symptom awareness warrant further evaluation in this context. This finding could be justified through multiple explanations. Ando et al. have shown that cardiopulmonary arrest (CPA) presentation is more prevalent among young adults, and its occurrence concomitant with acute MI in these patients accompanies a higher rate of in‐hospital mortality.^27^ Furthermore, an increased in‐hospital mortality rate is associated with arrhythmias, which are more prevalently seen among younger patients.^24^ In this regard, the differences in the pathogenesis of AMI between younger and older patients must also be taken into consideration. Although atherosclerotic plaque rupture accounts for the majority of events in both cohorts, etiologies such as plaque erosion, spontaneous coronary dissection, and vasospastic angina are more common among younger age groups and may contribute to the difference found in the in‐hospital mortality rate within groups.^28^ The higher risk of in‐hospital mortality in our study may also be partly explained by the fact that all the in‐hospital events in our study population occurred in men and those presenting with STEMI. This can be indicative of profound tissue destruction and more attenuated left ventricular (LV) systolic function in this patient field.^24^ As shown in previous studies, STEMI and decreased LV ejection fraction are independent predictors of short‐term adverse outcomes in young patients with ACS,^29^ thus, affecting our results in a way that premature in‐hospital mortality scored higher than that of the mature cohort.^30^, ^31^

### 4.3 Outcomes: Men versus women

Our unadjusted results suggested that both premature men and women have lower all‐cause mortality and MACCE. Yet after adjustment, only young women had significantly lesser all‐cause mortality. There is no consensus on sex differences in ACS. The contemporary studies, in contrast with previous ones,^32^ suggest no gender difference in long‐term mortality and MACCE.^33^ Sedlak et al. propose improved procedure rates and widespread medical management of ACS as plausible explanations for the mentioned discrepancy.^34^ As well as this, the prevalence of nonobstructive CAD is higher among young women, with spontaneous coronary artery dissection, coronary microvascular dysfunction, and coronary vasospasm underpinning ACS in these cases.^28^ Therefore, enhanced diagnosis and treatment of the aforementioned conditions may contribute to better outcomes in this subgroup.^34^ There were no cases of in‐hospital mortality among women, and no gender comparison could be conducted in this regard.

## 5 STUDY LIMITATIONS

Our study encompasses some limitations. First, we missed data on patients who passed away before reaching the hospital, with presumably more severe ACS as fatal coronary heart diseases are associated with a higher prevalence of risk factors field.^35^ Second, in the current study, the presence of risk factors such as HTN and DM was based on patient history and not documented criteria. Therefore, their prevalence may have been underestimated, particularly among younger patients, who probably have not undergone screening. Lastly, patients with known previous cardiovascular diseases may have altered their lifestyle or have received medication (e.g., lipid‐lowering therapy), modifying their profile of risk factors.

## 6 CONCLUSION

Risk factor distribution varies in age groups; while smoking, family history, and dyslipidemia are higher in young, DM and HTN are more frequent among older patients. Despite the general belief that considers lower event rates among the younger population, our results showed comparable MACCE and all‐cause mortality among premature and nonpremature ACS patients who underwent PCI. Hence, organizing strict secondary preventive measures specifically designed for given risk factors in each group would result in the most favorable outcomes regardless of the age of the first CAD event.

## CONFLICT OF INTEREST STATEMENT

The authors declare no conflict of interest.

## Data Availability

The datasets used and/or analyzed during the current study are available from the corresponding author upon reasonable request.
